# Comparing the performance of circulating cathodic antigen and Kato-Katz techniques in evaluating *Schistosoma mansoni* infection in areas with low prevalence in selected counties of Kenya: a cross-sectional study

**DOI:** 10.1186/s12889-018-5414-9

**Published:** 2018-04-11

**Authors:** Collins Okoyo, Elses Simiyu, Sammy M. Njenga, Charles Mwandawiro

**Affiliations:** Eastern and Southern Africa Centre of International Parasite Control, Kenya Medical Research Institute (KEMRI), P.O. Box 54840 – 00200, Nairobi, Kenya

**Keywords:** *Schistosoma mansoni*, Kato-Katz, Circulating cathodic antigen

## Abstract

**Background:**

Kato-Katz technique has been the mainstay test in *Schistosoma mansoni* diagnosis in endemic areas. However, recent studies have documented its poor sensitivity in evaluating *Schistosoma mansoni* infection especially in areas with lower rates of transmission. It’s the primary diagnostic tool in monitoring impact of the Kenya national school based deworming program on infection transmission, but there is need to consider a more sensitive technique as the prevalence reduces. Therefore, this study explored the relationship between results of the stool-based Kato-Katz technique with urine-based point-of-care circulating cathodic antigen (POC-CCA) test in view to inform decision-making by the program in changing from Kato-Katz to POC-CCA test.

**Methods:**

We used two cross-sectional surveys conducted pre- and post- mass drug administration (MDA) using praziquantel in a representative random sample of children from 18 schools across 11 counties. A total of 1944 children were randomly sampled for the study. Stool and urine samples were tested for *S. mansoni* infection using Kato-Katz and POC-CCA methods, respectively. *S. mansoni* prevalence using each technique was calculated and 95% confidence intervals obtained using binomial regression model. Specificity (Sp) and sensitivity (Sn) were determined using 2 × 2 contingency tables and compared using the McNemar’s chi-square test.

**Results:**

A total of 1899 and 1878 children were surveyed at pre- and post-treatment respectively. *S. mansoni* infection prevalence was 26.5 and 21.4% during pre- and post-treatment respectively using POC-CCA test, and 4.9 and 1.5% for pre- and post-treatment respectively using Kato-Katz technique. Taking POC-CCA as the gold standard, Kato-Katz was found to have significantly lower sensitivity both at pre- and post-treatment, Sn = 12.5% and Sn = 5.2% respectively, McNemar test χ^2^_m_ = 782.0, *p* < 0.001. In overall, the results showed a slight/poor agreement between the two methods, kappa index (k) = 0.11, *p* < 0.001, inter-rater agreement = 77.1%.

**Conclusions:**

Results showed POC-CCA technique as an effective, sensitive and accurate screening tool for *Schistosoma mansoni* infection in areas of low prevalence. It was up to 14-fold accurate than Kato-Katz which had extremely inadequate sensitivity. We recommend usage of POC-CCA alongside Kato-Katz examinations by Schistosomiasis control programs in low prevalence areas.

## Background

Existing guidelines by world health organization (WHO) for control and elimination of Schistosomiasis recommends baseline evaluations of the prevalence of Schistosome infections to inform programmatic decisions on target populations and treatment frequency within endemic areas [1]. In evaluating *Schistosoma mansoni*, mapping by examinations for parasite eggs in the stool using Kato-Katz thick smear microscopic technique is usually the practice [2]. This technique is usually preferred by control programmes in areas of moderate to high infection intensity and prevalence due to its ability to give both intensity and prevalence data for large sample of subjects. Due to its extensive use, it was established as part of WHO guidelines for morbidity control programmes [3]. The guideline recommends examining one stool per subject using two separate Kato-Katz slides read by two microscopists when estimating prevalence and intensity for control programmes, this is believed to provide near true prevalence estimate in high-mean intensity areas [4].

However, the Kato-Katz technique has showed day-to-day and intra-stool variability especially in areas with low intensity of infection and prevalence [5–7], with the cause of day-to-day variation believed to be biological [8], and the intra-stool variation attributed to sampling error due to the amount of stool evaluated [9]. This has rendered the technique less useful especially in areas with lower rates of transmission with many studies now projecting less true prevalence estimates from this technique as the population mean intensity of infection decreases [1, 10–13]. Programmes depending on single stool examination possibly lead to underestimated true infection prevalence with such observation resulting in an inaccurate allocation of control measures.

Owing to the reduced sensitivity of Kato-Katz technique in areas of low endemicity, improved diagnostic methods for accurate detection of *S. mansoni* in the at-risk populations and monitoring progress of control programmes are desirable. Several studies have now documented the availability of indirect diagnostic tests like point-of-care circulating cathodic antigen (POC-CCA) as valuable alternatives to the direct parasitological methods for diagnosis of *S. mansoni* [6, 14, 15]. This test detects Schistosome glycoprotein in the host urine after being deposited into the bloodstream by actively feeding worms and subsequently cleared from the host’s kidneys [14]. The Schistosome antigens; both CCA and circulating anodic antigen (CAA) are detectable in urine of infected individuals and they act as specific markers for the presence and intensity of infection [6, 16, 17]. Studies that have been conducted in Kenya [18], Uganda [15, 19], and Ivory Coast [16] to assess the performance of CCA urine dipstick and POC-CCA test among pre-school and school-aged children have both determined the usefulness of this technique in detection of *S. mansoni* infection in those age groups. These findings have been corroborated further by systematic reviews that have found POC-CCA to be up to 6-fold accurate than Kato-Katz technique especially in areas of low prevalence [5].

In Kenya, the ministries of health and of education began a national school-based deworming (NSBD) programme in the year 2012 in 27 counties identified as having a high prevalence of both soil-transmitted helminthes (STH) and Schistosome infections [20, 21]. According to the Kenyan National School Health Policy, treatment for both STH and Schistosome infections is administered to all school-aged children including those out of school, based on the baseline prevalence in the identified areas so as to reduce infection [21]. This national programme has been using Kato-Katz technique as the primary diagnostic tool in monitoring the impact of infection transmission over a five-year period (2012–2017). Having reduced the prevalence of *S. mansoni* infection over the five years, and due to the growing concern over the reduced sensitivity of Kato-Katz technique, this current cross-sectional study was therefore designed to compare the performance of the stool-based Kato-Katz technique with the commercially available urine-based POC-CCA test with the view to inform decision-making by the programme in changing from Kato-Katz to POC-CCA test in its effort to control transmission of Schistosomiasis.

## Methods

### Study design

This current study was nested within the ongoing five-year monitoring and evaluation (M&E) programme of the NSBD programme which utilized a series of pre- and post- intervention, repeat cross-sectional surveys in a representative, stratified, two-stage sample of schools across Kenya [20, 21]. However, our study used two cross-sectional surveys conducted during pre- and post-treatment at year five of that M&E programme. We used simple random sampling technique to randomly sample 18 schools out of the 200 schools currently being followed by the M&E programme, the included schools across 11 counties from Coastal, Rift Valley, Nyanza and Western regions of Kenya were based on their low *S. mansoni* prevalence of circa 10% (using Kato-Katz technique) at fourth year of that programme [22].

In each school, 18 children (9 girls and 9 boys) were sampled randomly from each of the six classes; one early childhood development (ECD) class and classes 2–6 using random number tables, for a total of approximately 108 children per school. Therefore, a random sample size of 18 schools with approximate total of 108 children per school was adequate to detect a 5% change in prevalence between the two methods while assuming a power of β = 0.8 and test size α = 0.05.

### Data collection

The pre-treatment surveys were conducted between 23rd January and 26th May, 2017, while post-treatment surveys were conducted between 13th March and 20th July, 2017 approximately 12–30 days after mass drug administration (MDA) to all school children by NSBD programme. Laboratory data reporting form was programmed onto android-based smart phones and used to capture data electronically into the open data kit (ODK) system [23], which included in-built data quality checks to prevent data entry errors.

### Survey procedures

#### Stool sample processing using Kato-Katz technique

Each participating child was asked to provide stool sample which was then processed in the laboratory within 24 h and examined in duplicate for the presence of *S. mansoni* eggs by two different technicians using the Kato-Katz thick-smear technique and prepared using a sieve and calibrated template of 41.7 mg, any resulting discrepancies were resolved by a third senior technician. For purposes of quality control, 10% of all stool samples were randomly re-examined by a senior technologist. The readings were recorded as number of eggs per gram of stool (EPG) for each child [24]. However, for purposes of this study, the *S. mansoni* infection was later simply categorized as being either positive or negative.

#### Urine sample processing using POC-CCA technique

Additionally, each participating child was also asked to provide urine sample which was processed in the laboratory within 24 h for the qualitative presumptive detection of an active *S. mansoni* eggs by the commercially available POC-CCA technique, according to manufacturer’s instructions (Rapid Medical Diagnostics, Pretoria) [25]. Two drops of urine (circa 80 μl) was transferred to the circular well of the test cassette and allowed to absorb entirely into the specimen pad, results were then read by two different technicians after 20 min after the assay had developed and any discrepancies examined by a third senior technician. The CCA tests were scored as negative, trace or positive (regardless of whether single or double band) based on formation and intensity of the control band [26, 27]. However, tests were considered invalid if an internal control band did not appear or if tests were not read after more than 25 min [1]. In those few cases, samples were re-run with a new cassette and scored as necessary. Tests were given a score of ‘trace’ if the control band was barely visible. In all our analysis herein, all ‘trace’ results were considered positive in agreement with van Dam et al. [26]. For quality control purposes, 10% of all urine samples were randomly re-examined by a senior technologist.

Both stool and urine samples were collected between 09:00 and 12:00 h each day of the survey. As part of the national school-based deworming programme, all participating children were treated with praziquantel for Schistosome infections according to WHO guidelines [24].

### Statistical analysis

#### Calculation of Kato-Katz parameters

True positive (TP) was defined as the number of both Kato-Katz and POC-CCA positive samples, true negative (TN) was the number of both Kato-Katz and POC-CCA negative samples, false positives (FP) were the number of Kato-Katz positive samples and POC-CCA negative samples, and false negatives (FN) were the number of Kato-Katz negative samples and POC-CCA positive samples. Therefore, the positive predictive values (PPV) were obtained using *TP*/(*TP* + *FP*)*x*100% whereas the negative predictive values (NPV) were obtained as *TN*/(*TN* + *FN*)*x*100%. Sensitivity (Sn) was calculated as *TP*/(*TP* + *FN*)*x*100% and specificity (Sp) was *TN*/(*TN* + *FP*)*x*100%. Under the current study, the performance of Kato-Katz thick smear method was evaluated against the POC-CCA technique as the reference test ‘gold standard’.

Kato-Katz performance was calculated and compared with the reference test, at 95% confidence intervals (CIs) for the following measures; Sn, Sp, PPV, NPV, positive likelihood ratio (LR^+^), negative likelihood ratio (LR^−^), and kappa score. Sensitivity and specificity were determined using 2 × 2 contingency tables and compared using McNemar’s chi-square test. PPV and NPV were determined using the weighted generalized score chi-square test for paired data [28]. Exact binomial 95%CI was calculated for each measure listed. Agreement between the two diagnostic methods was determined by calculating kappa statistics with 95%CIs with kappa values interpreted according Landis and Koch classification [29].

The overall prevalence of *S. mansoni* infection from each diagnostic method was calculated at the school and county level and the 95%CI obtained using binomial regression model.

All statistical analyses were carried out using STATA version 14.0 (STATA Corporation, College Station, TX, USA).

## Results

Overall, 1899 children with mean age of 9.7 years (2–18 years) were surveyed in the pre-treatment survey, and 1878 children with mean age 9.4 years were surveyed during the post-treatment survey as shown in Table 1. All the pre-treatment surveys were conducted approximately 17 days before the treatment day and post-treatment surveys conducted 12–30 days after the treatment delivery. Information on sex was recorded for 98.8% of the children out of which 50.4% were male. The study did not necessarily survey same children during both pre- and post-treatment surveys and those children absent on the day of the survey were not included in the study; moreover, children who did not provide both stool and urine samples were excluded from further analysis.

**Table 1:** Number of schools and children examined by county

County	Pre-treatment		Post-treatment	
	Number of schools	Number of children	Number of schools	Number of children
Bomet	1	108	1	103
Bungoma	1	108	1	105
Busia	2	214	2	210
Homa Bay	1	108	1	102
Kakamega	2	212	2	213
Kisii	1	105	1	103
Kisumu	3	312	3	313
Kwale	3	317	3	316
Mombasa	1	98	1	108
Narok	2	215	2	214
Taita Taveta	1	102	1	91
Overall	18	1899	18	1878

Table 2 compares the observed *S. mansoni* prevalence using the two techniques in overall and by county. The prevalence of *S. mansoni* infection was calculated with one Kato-Katz technique and compared with one POC-CCA technique. The observed prevalence using POC-CCA technique was 26.5% (95% CI: 24.6–28.6) during pre-treatment and 21.4% (95% CI: 19.6–23.4) during post-treatment compared to those observed when using Kato-Katz technique of 4.9% (95% CI: 4.0–5.9) and 1.5% (95%CI: 1.0–2.1) for pre- and post-treatment respectively. The observed prevalence for both pre- and post-treatment of *S. mansoni* infection using POC-CCA technique were significantly higher (χ^2^ = 135.58, *p* < 0.001) than those observed using Kato-Katz technique.

**Table 2:** Comparison of *S. mansoni* prevalence (%) using POC-CCA and Kato-Katz techniques among school aged children

County	Kato Katz			POC-CCA			Kappa statistics (Agreement %)
	Pre-treatment (%)	Post-treatment (%)	RR (%)	Pre-treatment (%)	Post-treatment (%)	RR (%)	
Overall	4.9 (4.0–5.9)	1.5 (1.0–2.1)	69.8	26.5 (24.6–28.6)	21.4 (19.6–23.4)	19.4	0.11 (77.1%)
Bomet	0	0	0	61.9 (53.3–71.9)	54.9 (46.0–65.5)	11.3	0.00 (42.1%)
Bungoma	0	2.0 (0.5–7.7)	+	8.6 (4.6–16.0)	14.6 (9.1–23.2)	+	0.06 (88.8%)
Busia	28.7 (23.2–35.5)	2.9 (1.3–6.4)	89.9*	46.6 (40.3–53.9)	23.3 (18.2–29.9)	50.0*	0.28 (71.4%)
Homa Bay	0	3.0 (1.0–9.1)	+	8.3 (4.5–15.6)	13.9 (8.5–22.5)	+	0.13 (89.3%)
Kakamega	1.9 (0.7–5.0)	5.8 (3.3–10.0)	+	20.2 (15.4–26.5)	36.8 (30.8–44.0)	+	0.12 (73.4%)
Kisii	0	0	0	12.1 (7.1–20.6)	15.0 (9.4–23.9)	+	0.00 (86.1%)
Kisumu	7.1 (4.7–10.6)	1.3 (0.5–3.5)	81.4*	34.3 (29.4–40.1)	14.1 (10.7–18.6)	58.9*	0.15 (77.4%)
Kwale	0	0	0	6.6 (4.3–10.2)	8.5 (5.9–12.3)	+	0.00 (92.5%)
Mombasa	1.9 (0.3–13.4)	0	100	9.7 (5.2–18.0)	5.6 (2.6–12.2)	42.1	−0.01 (90.3%)
Narok	0.9 (0.2–3.7)	0	100	47.4 (41.1–54.6)	42.9 (36.7–50.1)	9.5	0.01 (55.2%)
Taita Taveta	0	0	0	19.2 (12.8–28.8)	2.4 (0.6–9.3)	87.7	0.00 (88.5%)

*RR* indicates relative reduction in prevalence

+indicates an increase in prevalence rather than reduction

*indicates a significant relative reduction in prevalence, *p* < 0.05

The number of children who tested positive or negative for each of the diagnostic methods is shown in Table 3, with the results showing that among the 1761 (92.7%) samples examined during pre-treatment, 446 discrepancies were recorded (27 false positives and 419 false negatives), while only 370 discrepancies being recorded during post-treatment.

**Table 3:** Comparative evaluation of the POC-CCA and the Kato-Katz parasitological examination for the diagnosis of *S. mansoni* infection

Diagnostic technique		Kato-Katz stool examination								
		Pre-treatment			Post-treatment			Overall		
		Positive	Negative	Total	Positive	Negative	Total	Positive	Negative	Total
POC-CCA urine examination	Positive	60	419	479	20	363	383	80	782	862
	Negative	27	1255	1282	7	1409	1416	34	2664	2698
	Total	87	1674	1761	27	1772	1799	114	3446	3560

Taking POC-CCA as the gold standard, Kato-Katz significantly correctly identified only 80 out of 862 POC-CCA - positive *S. mansoni* infections; 9.3% Sn, (95% CI: 7.4–11.4; McNemar test = 782.0, *p* < 0.001) and 2664 out of 2698 POC-CCA – negative samples; 98.7% Sp, (95% CI: 98.2–99.1; McNemar test = 34.0, *p* < 0.001). The sensitivity of Kato-Katz was twice lower during post-treatment than pre-treatment (pre-treatment Sn = 12.5%, post-treatment Sn = 5.2%, *p* < 0.001). Overall, Kato-Katz resulted in a slight/poor detection of *S. mansoni* infection; k = 0.11, *p* < 0.001, concordance = 77.1% (Table 4). In all those counties i.e. Bomet, Kisii, Kwale and Taita Taveta, where *S. mansoni* prevalence was zero during both pre- and post-treatment by Kato-Katz technique, sensitivity of Kato-Katz was also found to be zero.

**Table 4:** Showing the performance measures of Kato-Katz by each survey round with POC-CCA as the gold standard

Treatment round	Sensitivity %(95% CI)	Specificity %(95% CI)	LR^+^ %(95% CI)	LR^−^ %(95% CI)	PPV %(95% CI)	NPV %(95% CI)	Kappa index (Agreement %)
Pre-treatment	12.5 (9.7–15.8)	97.9 (97.0–98.6)	6.0 (3.8–9.3)	0.9 (0.9–0.9)	69.0 (58.1–78.5)	75.0 (72.8–77.0)	0.14 (74.7%)
Post-treatment	5.2 (3.2–7.9)	99.5 (99.0–99.8)	10.6 (4.5–24.8)	1.0 (0.9–1.0)	74.1 (53.7–88.9)	79.5 (77.6–81.4)	0.07 (79.4%)
Overall *P*-value**	9.3 (7.4–11.4) Χ^2^_m_ = 782.0, *p* < 0.001	98.7 (98.2–99.1) Χ^2^_m_ = 34.0, *p* < 0.001	7.4 (5.0–10.92) -	0.9 (0.9–0.9) -	70.2 (60.9–78.4) -	77.3 (75.9–78.7) -	0.11 (77.1%) Z = 11.6, *p* < 0.001

*PPV* positive predictive value, *NPV* negative predictive value, *LR*^*+*^ positive likelihood ratio, *LR*^*−*^ negative likelihood ratio

**Obtained from McNemar’s chi-square (Χ^2^_m_) test (sensitivity & specificity) / Weighted generalized score chi-square test (PPV & NPV)

Suppose otherwise we take Kato-Katz as the gold standard, the overall POC-CCA sensitivity was found to be 70.2% and specificity was 77.3%, with higher sensitivity and specificity noted during post-treatment, (Sn = 74.1%, Sp = 79.5% respectively, and *p* < 0.001).

## Discussion

National Schistosomiasis control programmes need diagnostic techniques which are sensitive, specific, rapid and easy to perform at point-of-care. Kato-Katz technique has long been the mainstay test in *Schistosoma mansoni* diagnosis in endemic areas by most epidemiological studies [2, 14]. However, recent studies have since documented its poor sensitivity in evaluating *S. mansoni* infection thus making it less useful especially in areas with lower rates of transmission [5–7]. The low sensitivity can be attributed to the relatively small stool sample examined, fluctuations in daily egg excretion and the heterogeneous distribution of eggs within the stool sample [30–32]. This study provides the first rigorous assessment of the performance of Kato-Katz technique in comparison to POC-CCA in a national Schistosomiasis control programme in selected areas with lower transmission rates in Kenya.

The number of children infected with *S. mansoni* as determined by POC-CCA assay of one urine sample was found to be significantly higher than those determined by duplicate Kato-Katz thick smears of one stool sample and indeed 5-fold higher during pre-treatment and 14-fold higher during post-treatment, this finding is in agreement with previous studies [1, 14, 30, 33, 34]. In fact, a recent systematic review by Kittur et al., [5] noted that whenever *S. mansoni* prevalence was above 50% by Kato-Katz then Kato-Katz and POC-CCA results yielded essentially the same prevalence. However, whenever the prevalence is below 50% by Kato-Katz then the POC-CCA prevalence was between 1.5 and 6-fold higher and could increase further as prevalence by Kato-Katz decreased.

Out of the 862 POC-CCA positive samples, Kato-Katz classified 782 (90.7%) as negative, a scenario which can be explained by the reasons mentioned above. On the other hand, of all the negative results by POC-CCA, Kato-Katz classified only 34 (1.3%) as positive indicating a good specificity for Kato-Katz technique.

We noted that interpretation of the POC-CCA result band and inter-reader variability especially when the result is a ‘trace’ is an issue in the use of this technique, the same challenge had also been documented by other studies [35–37]. However, with better training in reading the result band, the challenge can be overcome. Other studies have suggested an addition of a comparison line on the assay to make reading of the result band easy and quick [34].

In comparison to one POC-CCA exam used as the gold standard, the stool-based Kato-Katz technique had extremely low sensitivity especially during post-treatment, but however had higher specificity both at pre- and post-treatment. In overall, the results demonstrated a significantly slight/poor inter-rater agreement between the two techniques; k = 0.11, *p* < 0.001, agreement = 77.1%. Our findings corroborate those of other studies done in Kenya [18], Uganda [15] and Ivory Coast [14] where POC-CCA tests detected *S. mansoni* infections in pre-school and school-aged at a higher sensitivity than the widely used Kato-Katz technique. Therefore, Kato-Katz and other direct diagnostic methods have inadequacies when it comes to accurate individual diagnosis which is further hampered by the fact that stools in young children are mostly diarrheic and renders the preparation of Kato-Katz thick smears difficult, hence challenging the accuracy of the diagnosis.

Finally, the study showed that even a single urine-based POC-CCA test seemed to be a more appropriate and effective screening tool for *S. mansoni* evaluation compared to stool-based Kato-Katz smears in areas with low infection prevalence. The tool was more sensitive and up to 14-fold accurate than Kato-Katz method. Although, POC-CCA test has known limitations like inter-reader variability in deciding a ‘trace’ result [30], more investigations can be conducted on how well the tool can distinguish between negative and ‘trace’ values. Even though the study found POC-CCA as a suitable alternative to Kato-Katz technique, it is known to provide limited intensity data and often no information on STHs infections [38], therefore we recommend its use for *S. mansoni* control programmes but with Kato-Katz for control programmes targeting both Schistosmiasis and STHs infections.

## Conclusions

Most large-scale Schistosomiasis control programs are based on preventive chemotherapy which usually reduces the infection prevalence and intensity of Schistosomes [39]. The frequent treatment normally results in lowered endemicity which goes hand-in-hand with reduced accuracy of Kato-Katz technique [40]. Hence, the need of a more sensitive and specific diagnostic tool for examination of *S. mansoni* after extensive mass treatment cannot be over-emphasized. This current study found POC-CCA method as more effective, and sensitive and it was up to 14-fold accurate than Kato-Katz. It was easy to use and less time consuming.

## Abbreviations

CAA: Cathodic anodic antigen
CIFF: Children’s investment fund foundation
CIs: Confidence intervals
ECD: Early childhood development
EPG: Eggs per gram
FN: False negative
FP: False positive
KEMRI: Kenya medical research institute
KNSBDP: Kenya national school-based deworming programme
LR^−^: Negative likelihood ratio
LR^+^: Positive likelihood ratio
M&E: Monitoring and evaluation
MDA: Mass drug administration
NPV: Negative predictive values
NSBD: National school-based deworming
ODK: Open data kit
POC-CCA: Point-of-care circulating cathodic antigen
PPV: Positive predictive values
Sn: Sensitivity
Sp: Specificity
STH: Soil-transmitted helminths
TN: True negative
TP: True positive
WHO: World health organization
